# Transient increase in international normalized ratio (INR) and bleeding risk following Alendronate sodium in elderly patients on warfarin: Two case reports

**DOI:** 10.1097/MD.0000000000018698

**Published:** 2020-01-10

**Authors:** Sufeng Qian, Jia Zhou, Pingda Bian, Lingfei Shi

**Affiliations:** a Department of Geriatrics, Diagnosis and Treatment Center of Osteoporosis, ZheJiang Provincial People's Hospital, People's Hospital of Hangzhou Medical College; b Department of Neurosurgery & Gamma Knife Center, Zhejiang Provincial People's Hospital & People's Hospital of Hangzhou Medical College, Hangzhou, Zhejiang, China.

**Keywords:** aged male, alendronate sodium, atrial fibrillation, osteoporosis, warfarin

## Abstract

**Introduction:**

Alendronate sodium is used to reduce the risk of bone fracture in aged osteoporosis patients. However, its side effects should be recognized, especially for those aged patients with one or more basic cardiovascular diseases.

**Patient concerns:**

A 90-year-old and a 75-year-old male patient were admitted to our department. These 2 patients were examined by dual energy X-ray absorptiometry (DXA).

**Diagnosis:**

Both patients were diagnosed with osteoporosis, they also had history of atrial fibrillation (AF) and had long term use of warfarin.

**Interventions:**

Alendronate sodium was prescribed to the two patients at 70 mg once a week.

**Outcomes:**

The 2 patients had experienced dramatic increase of international normalized ratio (INR) to 4.69∼4.86 within 24 hours and gradual decrease in the next 5 days. Both patients experienced spontaneous ecchymoses and petechiae in the skin at the first 72 hours.

**Conclusion:**

Alendronate sodium can transiently increase the INR over 50%, induce spontaneous ecchymoses and petechiae in the skin of aged male osteoporosis patients with AF who took warfarin. Clinicians should pay enough attention when using alendronate sodium on these kinds of patients and be aware of the consequent potential bleeding risk.

## 1 Introduction

Alendronate sodium is one of the most commonly used bisphosphonates for aged osteoporosis patients in China. This medication has high affinity with hydroxyapatite in bone union, specifically combined to the bone surface where active bone transformation taken place. Meanwhile, it could inhibit osteoclast function, reduce bone resorption, reduce the risk of bone fracture in centrum, hip and other parts of skeleton.^[1,2]^ Recently, we found that not only international normalized ratio (INR) of coagulation had been upregulated, but spontaneous ecchymoses and petechiae in the skin had also been caused after the administration of alendronate sodium in 2 elderly male atrial fibrillation (AF) patients who received warfarin anticoagulation therapy. To our knowledge, this side effect of alendronate sodium has not been reported before.

## 2 Case report

### 2.1 Case 1

A 90-year-old male diagnosed with osteoporosis after examined by dual energy X-ray absorptiometry (DXA) was admitted into our department. His serum carboxy-terminal cross-linking telopeptide of type 1 collagen (CTX), procollagen type 1 amino-terminal propeptide (P1NP) and osteocalcin (OC) was 0.73 μg/L (reference value 0.4 μg/L-0.78 μg/L), 104.80 μg/L (reference value 9.06 μg/L-76.24 μg/L), and 34.30 μg/L (reference value 6.00 μg/L-24.66 μg/L), respectively. He had a history of hypertension for over 20 years, chronic AF for 1 year. Six months earlier he suffered from sudden sensation of weakness in the right limb and slurred speech when woke up in the morning, and the brain MRI indicated ‘scattered lacunar infarction foci in right frontal lobe, infarction foci in left dorsal thalamic, malacic foci in right occipital lobe’. From then on, he took warfarin sodium tablet (produced by Orion Corporation in Finland) 2 mg/day. The patient's INR was 3.04 when he was admitted into our department. Oral alendronate sodium tablets (produced by Merck Sharp & Dohme Italia SPA Australia) once a week at 70 mg were prescribed to the patient. Surprisingly, 24 hours after the first dose, the patient's serum INR increased from 3.04 to 4.86, the oral taken of alendronate sodium tablets was stopped immediately. Then the patient's serum INR fell to 4.41 at 48 hours, meanwhile, he experienced spontaneous ecchymoses and petechiae in the skin. Fortunately, the ecchymoses and petechiae stopped progressing on the third day, and INR gradually decreased to 3.67 on the fifth day. The patient denied taken other medication except for warfarin sodium and alendronate sodium tablets, he also denied significant diet change.

### 2.2 Case 2

The patient that admitted into our department was a 75-year-old male, diagnosed with osteoporosis by DXA examination, his serum CTX, P1NP and OC were 0.78 μg/L, 200.70 μg/L, and 24.47 μg/L, respectively. He had a history of 15-year hypertension and 10-year chronic AF. Ten months earlier, he suffered from abrupt dizziness accompanied with nausea and vomiting, right limb dysfunction when he went to the toilet at night. CT scans showed ‘the left basal ganglia infarction foci’. After that, warfarin sodium tablet was orally taken 3 mg/day. The patient's INR was 2.70 when he was admitted into our department. Alendronate sodium tablets were prescribed to the patient at 70 mg once a week. It turned out that the patient serum INR increased from 2.70 to 4.69 at the first 24 hours, the oral taken of alendronate sodium tablets was stopped immediately. Then the patient's serum INR fell to 4.34 at 48 hours; he also had spontaneous ecchymoses and petechiae in the skin during the fluctuation of INR. However, the ecchymoses and petechiae gradually vanished at the following days and INR decreased to 3.32 at the fifth day. The patients denied taken other medication except for warfarin sodium and alendronate sodium tablets, he also denied significant diet change.

## 3 Discussion

In the elderly people, the prevalence of AF is as high as 6% and increases with age. The most serious complication of AF is thromboembolism of brain vessels, which can induce acute cerebral ischemic apoplexy and jeopardize patient's life.^[3]^ Therefore, long-term anticoagulation therapy using warfarin to maintain INR at 2-3 was a common method for these patients.

After stroke, elderly male patient's bone loss would significantly accelerated and bone density decreases rapidly due to prolonged bed time, reduced standing and walking, shortened sunshine bath time, and the influence of some drugs (such as hormones).^[4]^ The increased serum level of bone turnover markers (BTMs) has been observed in these patients, such as CTX, P1NP, and OC. Both CTX and P1NP are related to the metabolism of type 1 collagen, P1NP is a metabolite of type 1 procollagen (synthesized and secreted by osteoblasts) that is lysed by polypeptidase, while CTX is the product of type 1 collagen lysed by the activity of osteoclasts. OC is a bone-specific 5–8KD protein produced by osteoblasts, and is the most common protein (about 3%) in bones except type I collagen (90%).^[5]^ Clinical studies have shown that CTX, P1NP and OC are moderate positively correlated, and all of them are negatively correlated with bone density.^[6]^ Therefore, the serum level of BTMs should be paid attention to and monitored after the ischemic stroke of aged patients, and anti-bone resorption drugs like alendronate sodium should be used for patients with rapid increasing or high level of BTMs.

According to these 2 cases, it could be inferred that alendronate sodium would enhance the anticoagulant effect of warfarin, and increase the INR on the next day over 50%. Warfarin is a substance containing the basic structure of 4-hydroxycoumarin, which binds to plasma protein over 99% after absorption, but its anticoagulant effect is affected by a variety of environmental factors (such as drugs and food).^[7]^ The mechanism of alendronate sodium enhancing the anticoagulation effect of warfarin may be:

(1) alendronate sodium can compete with warfarin to bind to plasma protein, thus increasing the concentration of free warfarin in blood^[8]^;
(2) warfarin is metabolized in the liver by cytochrome P450–2C9 enzyme (CYP2C9) and vitamin K epoxide reductase (VKORC1), while alendronate sodium and its metabolites may inhibit the activity of CYP2C9 and VKORC1, thereby reducing the metabolism of warfarin^[9]^;
(3) alendronate sodium is a powerful inhibitor of osteoclast activity, which can cause transient decrease of serum calcium after taking, affect the blood coagulation process, extend the prothrombin time, and lead to transient increase of INR.^[10]^

Although the increased INR can fall to a lower level in a few days as well as the spontaneous ecchymoses and petechiae in the skin tend to self-cured after the administration of alendronate sodium, enough attention to the potential bleeding risk should be paid when using this medication. In order to reduce these potential risks for elderly male AF patients that received warfarin anticoagulation therapy who also need to take alendronate sodium, the following advices may be helpful:

(1) before taking alendronate sodium, patient's INR should be rechecked. If the INR is over 3 or fluctuates greatly, the administration of alendronate tablets should be suspended;
(2) on the day of taking alendronate sodium, for those patients with ideal and stable INR, the dose of warfarin should be appropriately reduced, the changes in INR as well as the appearance of ecchymoses and petechiae in the skin should be monitored;
(3) choose new type anticoagulation medication such as Rivaroxaban be the alternative of warfarin.^[11]^

## 4 Conclusion

Alendronate sodium can transiently increase the INR over 50%, induce spontaneous ecchymoses and petechiae in the skin in aged male osteoporosis patients with AF who had long term oral administration of warfarin. The accurate mechanism of this side effect of alendronate sodium in specific patients and whether other bisphosphonates (such as zoledronic acid, etc) would also interference the anticoagulant effect of warfarin need further studies.

## Author contributions

**Conceptualization:** Lingfei Shi.

**Funding acquisition:** Jia Zhou, Pingda Bian.

**Resources:** Lingfei Shi.

**Supervision:** Lingfei Shi.

**Writing – original draft:** Sufeng Qian, Jia Zhou.

**Writing – review & editing:** Sufeng Qian, Jia Zhou, Lingfei Shi.
